# Bilayer-Embedded Lipid Droplets Coated with Perilipin-2 Display a Pancake Shape

**DOI:** 10.3390/ijms24032072

**Published:** 2023-01-20

**Authors:** Sevde Puza, Shima Asfia, Ralf Seemann, Jean-Baptiste Fleury

**Affiliations:** Experimental Physics and Center for Biophysics, Saarland University, 66123 Saarbrücken, Germany

**Keywords:** lipid droplet, diffusion, FRAP, phospholipid, ADRP

## Abstract

Lipid droplets (LD) are organelles localized in the membrane of the endoplasmic reticulum (ER) that play an important role in many biological functions. Free LDs that have been released from the ER membrane and are present in the cytosol resemble an oil-in-water emulsion. The surface of an LD is coated with a phospholipid monolayer, and the core of an LD is composed of neutral lipids. Adipose differentiation-related protein (ADRP), also known as perilipin-2, is a protein that surrounds the LD, together with the phospholipid monolayer. ADRP molecules are involved in assisting in the storage of neutral lipids within LDs. In this article, we focus our interest on the influence of ADRP molecules on the 3D shape of bilayer-embedded LDs and the diffusion of phospholipids in the monolayer covering LDs. For this study, we employed two different microfluidic setups: one to produce and explore bilayer-embedded LDs and a second one to mimic the surface of a single LD. Using the first setup, we demonstrate that ADRP molecules stay preferentially localized on the surfaces of bilayer-embedded LDs, and we study their 3D-shape in the presence of ADRP. Using the second setup, we performed FRAP experiments to measure the phospholipid diffusion on a model LD surface as a function of the ADRP concentration. Although the presence of proteins on the LD surface minimally affects the phospholipid and protein motility, ADRP appears to have a significant effect on the 3D structure of LDs embedded in the bilayer.

## 1. Introduction

In recent years, lipid droplets (LDs) have attracted attention due to their roles in several diseases, metabolic disorders, and atherosclerosis [1,2,3]. LDs are organelles that are produced by the endoplasmic reticulum (ER) membrane [4,5] and are composed of a core made of neutral lipids, such as triglycerides and sterol esters, surrounded by a phospholipid monolayer [4,5]. The origin of their biogenesis is still under debate [6]. However, it is generally accepted that their biogenesis occurs in three steps [6,7]. First, neutral lipids are synthesized in the ER membrane [8]. In a second step, these neutral lipids diffuse in the membrane and agglomerate to produce nanometer-size oil inclusions. These nanometric hydrophobic inclusions are LDs that are supposed to have an initial diameter of approximately 50 nm [9,10]. The growth and enlargement of these LDs are caused by the ongoing production of neutral lipids inside the ER membrane. The LDs can spontaneously bud in the ER membrane as they grow, and are released into the cytosol when they pinch off from the membrane in the final step [11]. After release, the LDs can be observed via fluorescent microscopy, or by transmission electron microscopy. It appears that LDs are relatively spherical, having diameters ranging from 0.1 to 5 to 100 μm, depending on the cell type [12]. Aside from phospholipids, the surfaces of these LDs are decorated with either peripheral proteins or integral membrane proteins that adopt a monotopic topology and that are involved in metabolic and other biological functions [13]. Proteins of the perilipin family are known to be associated with the surfaces of LDs [14]. Adipose differentiation-related protein (ADRP or perilipin-2), is a lipid droplet protein from the perilipin family that is found in most cells and tissues [15]. Perilipin controls important intracellular lipases, although to a very different degree [16]. Perilipin-2 has been proposed to play a role in LD budding [17]. Studies on the subcellular localization of LDs suggest that the budding of LDs from the ER may take place in perilipin2-enriched domains in the ER membrane [17,18]. This is a crucial step, as the budding step precedes the release of the LDs into the cytosol, where they can transport material to other organelles in the cell [19,20].

The full-length perilipin-2 protein is made of 437 amino acids and is self-assembled in several α-helices. Its secondary structure available from the AlphaFold openIA database [21]. It is supposed that ADRP plays a key role in the management of neutral lipid stores [15,22]. How perilipin-2 proteins target and bind to lipid droplets (LDs) is poorly understood, even if alpha helices seem to play a key role in the targeting mechanism [23,24]. In living cells or in artificial LDs, ADRP has been found to barely affect the phospholipid diffusion properties [25].

In this manuscript, we investigate the effects of ADRP on phospholipid diffusion and the 3D shape of lipid droplets embedded in a bilayer. For that, we used two different microfluidic setups: One for the fabrication of bilayer-embedded LDs and a second to mimic the surface of a single LD [26,27]. Using the first setup, we found that ADRP remains preferentially localized on the surfaces of bilayer-embedded LDs and investigated the impact of ADRP on the 3D geometry of bilayer-embedded LDs. In addition, we measured the effect of ADRP concentration on the exchange rate of phospholipid molecules between the monolayer and bilayer [26] by performing fluorescent recovery after photobleaching (FRAP) experiments. With the second setup, we determined the phospholipid diffusion on an artificial LD’s surface as a function of ADRP concentration [27] by FRAP measurements.

## 2. Results and Discussion

### 2.1. LD Insertion and ADRPs’ Localization in a Free-Standing Bilayer

**Figure 1:**
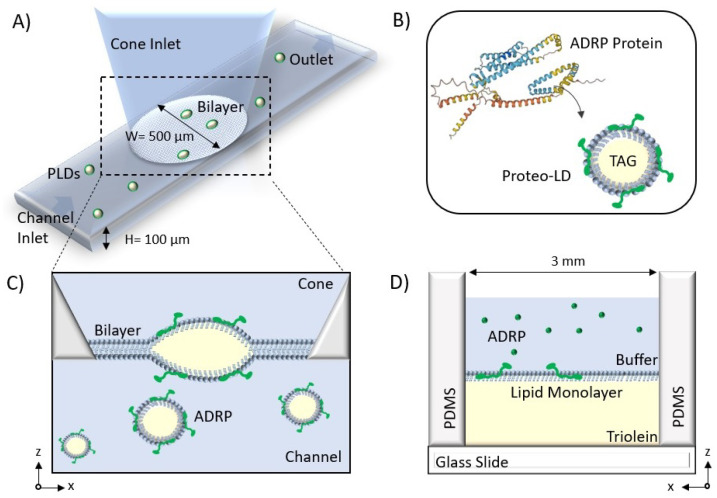
(**A**) Schematic of the microfluidic system to form a free-standing bilayer containing Proteo-LDs. (**B**) The secondary structure of ADRP was provided by the AlphaFold AI database. ADRP is reconstituted in the PC:PE monolayer covering the LD. (**C**) A bilayer is formed at the aperture between the channel and the cone, and LDs containing ADRP protein are introduced from the bottom channel and insert into the bilayer. (**D**) Schematic of the microfluidic device to form the free-standing monolayer. A phospholipid monolayer is formed at the buffer–triolein interface.

**Figure 2:**
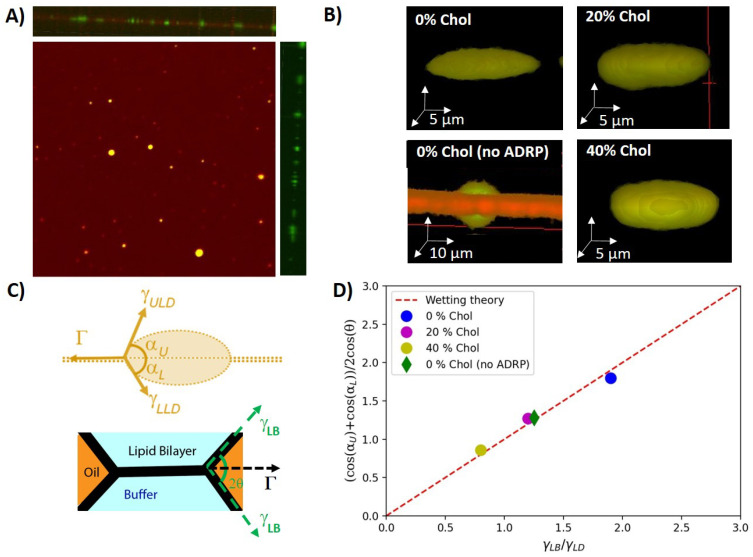
(**A**) Top view of a free-standing bilayer containing LDs. A side view of a 3D scan is provided in the top panel. The right panel provides the corresponding side view with only the protein channel. All the proteins are labeled in green (Alexa 488) and 2% (molar ratio) DOPE is labeled in red (Cy5). (**B**) 3D reconstructions of individual bilayer-embedded LDs for 0%, 20%, and 40% cholesterol, respectively (with 1 μM ADRP). For comparison, the 3D reconstructions of a bilayer-embedded LD with 0% cholesterol are given, and no ADRP was added. In the latter case, the neutral lipids were stained with BODIPY. (**C**) Scheme of the surface forces acting on a bilayer-embedded LD with an ideal lens shape (upper panel). Scheme of the surface forces acting on the bilayer (lower paner). (**D**) Comparison between wetting theory and the balance of surface forces as extracted from the symmetric 3D LD geometry.

We employed a 3D microchip [26] that enables the formation of a free-standing horizontal lipid bilayer at a desired position (see Figure 1, as detailed in the Section 3). The bilayer consisted of DOPC:DOPE with a molar ratio 3:1, which can be considered as a model endoplasmic reticulum membrane [28]. After lipid bilayer formation, we dispensed protein containing LDs through the bottom channel of the chip. From now on, LDs always refer to lipid droplets enriched with some high concentration of ADRP protein, if not mentioned otherwise (see Section 3). Due to their preparation, the LDs were normally about 15 μm in size. Fluorescent visualization of the LDs in buffer revealed that the protein was present throughout the entire LD, and not only on the surface. The same observation was made for several type of LDs coated with perilipins [23,24,25]. The estimated diffusion coefficient of a freely dispersed LD of about 15 μm is ≈0.03–0.04 μm${}^{2}$·s${}^{-1}$ [29]. This diffusion coefficient is sufficient for the LDs to diffuse freely and to finally reach the lipid bilayer. A LD in contact with a bilayer can insert into the bilayer core, creating an inclusion with a distinctive shape [26,30], and stops moving [26]. This insertion into the bilayer was verified by 3D confocal scans; see Figure 2. The absence of any detectable fluorescent signal from ADRP in the bilayer demonstrates that the proteins stay localized in and on the bilayer-embedded LD. However, it might still be possible that some negligible amounts of protein move in the bilayer. To test this, we bleached the proteins present in the core and on the surface of a single bilayer-embedded LD and waited 1 h for any fluorescent protein signal to recover. However, no fluorescence recovery was observed during that time, indicating that there is in fact neither ADRP in the bilayer nor any measurable protein transport between different bilayer-embedded LDs. This point is interesting, as it was also observed in living cells [25].

### 2.2. Influence of ADRP on the Shape of Bilayer-Embedded LD

Having inserted LDs into a free-standing lipid bilayer, we explored their 3D shape in view of the acting surface forces. The LDs were consequently divided into two groups: tiny drops and pancakes. Tiny droplets are too small to have their geometry analyzed in detail. In consequence, we ignored these tiny droplets in the following analysis. Pancake droplets have a diameter of about 20 μm and a height of about 5 μm, which was sufficiently large to resolve that their shapes escaped slightly from the expected ideal lens shape with spherical interfaces (Video S1). LDs without proteins, however, revealed a larger contact angle, and the shape of the two lens surfaces was spherical; see Figure 2B and Table 1 [10,30,31]. Indeed, surface-tension measurements revealed that ADRP is highly surface active. For example, a triolein–buffer interface with 1 μM of ADRP, directly dispersed into the buffer, revealed a surface tension of ≈10 mN/m, which is only one third of the surface tension of a triolein–buffer surface (≈30 mN/m) not containing and surface active molecules (as determined by pendant-droplet method). Thus, the reduced contact angle and the slightly flattened LD shape with ADRP molecules being present of the LD surface might have resulted from an inhomogeneous distribution of the surface active ADRP molecules (see Section 3 and Table 1), which might have an increased presence close to the circumference of the LD, lowering the local surface tension.

To check the shape of the LDs’ and their influence on the wetting properties in some more detail, we measured the shapes of the LDs with bilayers having different cholesterol concentrations. The usage of cholesterol is not biorelevant, as ER membranes do not contain cholesterol. However, it allowed us to tune the surface forces applied to the LDs [26]. Interestingly, cholesterol appears to increase the LD surface tension as measured by the pendant-droplet method and also increased the contact angle of the LDs (see Table 1). However, the pancake-like shape in the presence of ADRP molecules remained and seemed even more pronounced; the larger the cholesterol concentration, the larger the contact angle α. This may suggest that the inhomogeneous distribution of ADRP on the LD’s surface is even enhanced in the presence of cholesterol.

The shape of embedded LDs is defined by the balance between the bilayer tension Γ and the tensions applied by the horizontal components of the surface tensions of the upper LD leaflet $\Gamma_{\mathrm{ULD}}=\gamma_{\mathrm{ULD}}\cdot \cos\left(\alpha_{U}\right)$ and the lower leaflet $\Gamma_{\mathrm{LLD}}=\gamma_{\mathrm{LLD}}\cdot \cos\left(\alpha_{L}\right)$ close to the three-phase contact line, such as:

(1) $$\Gamma =\gamma_{\mathrm{ULD}}\cdot \cos\left(\alpha_{U}\right)+\gamma_{\mathrm{LLD}}\cdot \cos\left(\alpha_{L}\right).$$

where $\alpha_{U}$, $\alpha_{L}$ are the angles measured from the 3D images, as defined in Figure 2C. In equilibrium, the bilayer tension can be determined by the Young–Dupré law, $\Gamma =2\gamma_{\mathrm{LB}}\cdot \cos\left(\theta \right)$ [31], where $\gamma_{\mathrm{LB}}$ is the surface tension of a monolayer-decorated oil–water interface and 2θ is the contact angle of the plateau border; see Figure 2C (measured by scanning directly the contact angle via confocal microscopy). As ADRP is partially soluble in the core of the LD, after a short equilibration time, it can be assumed that the amount of proteins at the interface of the LDs and thus the surface tension of both droplet interfaces are equal on average. As a reasonable simplification, we can thus assume $\gamma_{\mathrm{ULD}}=\gamma_{\mathrm{LLD}}=\gamma_{\mathrm{LD}}$ and reformulate Equation (1) to

(2) $$\frac{\gamma_{\mathrm{LB}}}{\gamma_{\mathrm{LD}}}=\frac{\cos\left(\alpha_{U}\right)+\cos\left(\alpha_{L}\right)}{2\cos(\theta )},$$

From the independently measured angles α and θ and the measured surface tensions values $\gamma_{\mathrm{LB}}$ and $\gamma_{\mathrm{LD}}$ (see Table 1), we could verify that these values satisfy Equation (2), as plotted in Figure 2D. It results that the measured pancake shapes correspond to an equilibrium wetting morphology, and the reduction of the insertion angle with increasing cholesterol concentration appears to be a consequence of increased bilayer tension as a function of increasing cholesterol concentration, which is consistent with some literature [32,33]. It also indicates that ADRP proteins qualitatively change the surface properties of bilayer-embedded LDs, as they do not exhibit a lens-like shape but a pancake shape (see Figure 2). In view of the quantitative agreement of the measured LD shape with bulk surface-tension measurements, it can be even assumed that the LD surface remote from the three-phase contact line is depleted of ADRP molecules.

### 2.3. Effect of ADRP on the Phospholipid Diffusion Properties of an LD Embedded in a Bilayer

In this section, we study the phospholipid diffusion properties in the presence of ADRPs. In this context, there are two aspects of particular interest to us: the influence of ADRP on the phospholipid exchange rate between the LD monolayer and the bilayer (in the case of a bilayer-embedded LD) and the influence of phospholipid diffusion on the LD monolayers themselves.

**Figure 3:**
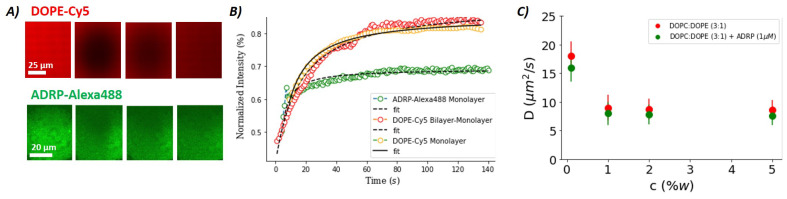
(**A**) Series of fluorescence images showing a lipid monolayer (DOPC:DOPE, 3:1) between buffer and triolein before bleaching and during recovery. (Upper panel) DOPE-Cy5, 2% molar ratio; (Lower panel) ADRP-Alexa488, 1 μM. (**B**) FRAP curves extracted from fluorescence images, such as the one shown in (**A**) with the corresponding Soumpasis fits for DOPE-Cy5 (red dots) and ADRP-Alexa488 (green dots). Yellow dots correspond to the recovery of the DOPE-Cy5 fluorescence obtained by bleaching the entire bilayer-embedded LD surface, with same bilayer composition as in (**A**). (**C**) Diffusion coefficients of DOPE in the lipid monolayer, as a function of total lipid concentration and in the presence and absence of ADRP. The error bars are presented by the continuous lines. In (**A**–**C**), each value was obtained by averaging ≈ 30 different measurements.

We begin our discussion by studying the phospholipid diffusion on an artificial LD surface. For this purpose, we realized a model LD surface using the microfluidic setup described in Figure 1D. The model LD surface was covered with a phospholipid monolayer at the buffer–triolein interface composed of DOPC:DOPE with a molar ratio of 3:1. As DOPE contains 2 w% of the fluorescent phospholipid DOPE-Cy5, we can perform FRAP experiments on this monolayer (see Figure 3) [27]. The diffusion coefficient extracted from the FRAP data is ≈8 μm${}^{2}\cdot$s${}^{-1}$ when the total phospholipid concentration in triolein used to form the monolayer is equal or above 1 w%; see Figure 3 [34]. Smaller phospholipid concentrations lead to an increased diffusion constant, which is expected for a monolayer with incomplete surface coverage. When these experiments were repeated with 1 μM ADRP, we observed that the ADRP molecules were slightly inhomogeneously distributed and seemed to cluster, showing granularity on the length scale of about 1 μm and 5 μm in fluorescent images. This indicates that the ADRP proteins do not remarkably affect the motility of a phospholipid monolayer. This feature seems to be consistent with results reported in the literature [23]. We also performed FRAP measurements on fluorescently labeled (ADRP-Alexa488) proteins (c ≈ 1 μM) present on the lipid monolayer. The thus extracted ADRP diffusion coefficient is ≈0.3 μm${}^{2}\cdot$s${}^{-1}$ when the total phospholipid concentrations used to form the monolayer were equal or above 1%w. This diffusion coefficient was more than 20 times smaller than that of the phospholipids, indicating slow motility of the proteins on the LD surface; however, this motility is still not negligible. Interestingly, to the best of our knowledge, this manuscript offers the first measurements of ADRP diffusion in a lipid monolayer.

The diffusion coefficient of PE in a bilayer is about 10 μm${}^{2}\cdot$s${}^{-1}$ [26]—i.e., very close to the diffusion coefficient of about 8 μm${}^{2}\cdot$s${}^{-1}$ measured for a densely packed DOPC/DOPE monolayer with or without ADRP present in the monolayer. However, despite the similar motilities in the bilayer and monolayer, it was found in previous experiments that a diffusion barrier between monolayer and bilayer exists in this system [26], which reduces the molecular transport rate between the bilayer and the monolayer. This was shown in [26] by the cone-shaped DOPE lipids, which are able to stabilize the negative membrane curvature along the LD edge and provide a structural source for the diffusion barrier, in line with other recent studies [35,36,37].

To test the possible existence of such a diffusion barrier also in the presence of ADRP molecules, we performed FRAP experiments on bilayer-embedded LDs; see Figure 3B. For that, we fabricated a free-standing bilayer composed of DOPC:DOPE with a molar ratio of 3:1 and inserted ADRP-enriched LDs into the bilayer, as was described previously. We bleached the fluorescent phospholipids (DOPE-Cy5) on the entire surface of the LD and measured the fluorescence recovery due to the transport of fluorescent phospholipids coming from the bilayer. Surprisingly, in the presence of ADRP, we measured that the DOPE-Cy5 fluorescence recovery curve is very similar to the one measured in a phospholipid mono- or bilayer. This result indicates that the presence of ADRP disassembles the hypothetic metastable PE ring and facilitates the molecular transport between the bilayer and the monolayer. This finding agrees perfectly with a preferential presence of ADRP molecules near the three-phase contact line, as already suspected based on the pancake-like shape of the LDs in the presence of ADRP molecules.

## 3. Materials and Methods

### 3.1. Molecules

1,2-Dioleoyl-sn-glycero-3-phosphocholine (DOPC), 1,2-dioleoyl-sn-glycero-3-phosphoe thanolamine (DOPE), and cholesterol were purchased from Avanti Polar Lipids. Glyceryl trioleate (triolein, T7140) and squalene (S3626) were purchased from Sigma-Aldrich. BODIPY (493/503, D3922) was purchased from Thermo-Fisher. Recombinant human denaturated full length perilipin-2 protein (ADRP) was purchased from Abcam (ab181932). We purchased (DOPE-Cy5.5)1,2-dioleoyl-sn-glycero-3-phosphoethanolamine-N-(cyanine 5.5) from Avanti Polar Lipid. Adipose differentiation-related protein (ADRP) is turned fluorescent by the Alexa Fluor 488 conjugation kit (Abcam-ab236553), following the procedure described in the next section. In this manuscript, buffer solution refers to 150 mM potassium chloride.

### 3.2. ADRP Protein Conjugation

Prior to conjugation, a stock solution with a concentration of 0.5 mg/mL was prepared by mixing the recombinant human ADRP protein with ultra-pure water (Thermo Fisher, Waltham, MA, USA). To label the proteins with the Alexa Fluor 488 conjugation kit (Abcam-ab236553), 5 μg of the proteins was used. For that, first, 1 μL of the modifier agent was added to 10 μL of the ADRP stock solution (buffer) and mixed gently. The mixed sample was pipetted into the reagent. This solution was kept standing in a dark room for 15 min, before mixing with 1 μL of the quenching agent. The conjugated proteins were ready to use after 5 min.

### 3.3. Free-Standing Bilayer Formation

A sketch of the 3D microfluidic device is shown in Figure 1A). The device consists of a horizontal channel at the bottom with an attached truncated cone and was fabricated in PDMS (Sylgard 184, Dow Corning) from a 3D mold following soft-lithographic protocols. Details of the design and the fabrication of the microfluidic 3D device can be found in [26].

We employed a bilayer with a constant DOPC:DOPE composition of 3:1 (unless mentioned otherwise), which can be considered as a model endoplasmic reticulum membrane [26,37]. All the phospholipids were prepared with a concentration of 5 mg/mL in squalene oil. The lipid–oil solution was mixed at 50 °C for 3–4 h to allow the lipids to dissolve completely in the oil. The bilayer formation in the 3D microfluidic device was done following three steps. First, the bottom channel was filled completely with the buffer solution. The height of the buffer was set so that its meniscus was located at the aperture formed by the intersection of the bottom channel and the upper cone. Then, gently, a 4 μL drop of lipid–oil mixture was pipetted into the upper cone, whereby the entire meniscus was covered with the lipid–oil mixture to form a first water–oil interface. Subsequently, a 5 μL drop of buffer was pipetted into the cone to cover the lipid–oil mixture and to form a second water–oil interface. The phospholipids cover the water–oil interfaces and form two separated phospholipid monolayers. At the same time, squalene is adsorbed by the PDMS, eventually bringing the two separate phospholipid monolayers into contact and forming a bilayer. Depending on the amount of the lipid–oil mixture trapped between the two buffer phases, the time required for the formation of a bilayer varies from 10 min to 2 h. When the formation of the bilayer begins, a small circle appears in the center of the aperture. This circle enlarges and eventually fills the entire aperture, which is the sign of complete zipping of the two monolayers and the formation of the bilayer [38]. All experiments were performed at a room temperature of 23 °C.

### 3.4. LD Preparation

Phospholipids (DOPC and DOPE) and triolein were used from their stock solution (10 mg/mL). In total, 30 μL of triolein with DOPC:DOPE (1:1 in molar ratio, and 2% of phospholipid in total weight ratio) was dried in a glass falcon under vacuum for 1 h. The composition of the phospholipid monolayer on the LD surface was chosen to allow easy LD insertion inside the free-standing bilayer (see [26]). For one experiment detailed in Figure 2B, showing a LD without ADRP, BODIPY was added to triolein to stain neutral lipids. Then, 1 μM of the conjugated protein solution was diluted in 10 μL PBS. This triolein–lipid mixture and 200 μL PBS were added to a falcon tube and stored at 4 °C overnight. Afterwards, the mixture was sonicated for 5 min to obtain LDs. The sizes of the obtained LDs were measured to be round 15 μm by optical microscopy. We injected 1 μM of ADRP into the LD dispersion and let them find and bind to the LD by diffusion. In summary, the formed LDs were coated with a PC:PE monolayer (molar ratio 1:1) containing ADRP proteins. Then, the LDs were dispersed around the bilayer by flowing them into the bottom channel of device 1; cf. Figure 1A. After (10–15) min, the spontaneous insertion of several of these LDs into the bilayer was observed using confocal microscopy [26].

Finally, to demonstrate the presence of ADRP on the LD surface, we repeated the formation of LDs without the use of phospholipids. Thus, we produced an emulsion of triolein in water with only ADRP as an emulsifier. The resulting triolein droplets were stable for at least of a few hours and presented a size in the range of 50–100 μm. This finding would be impossible to achieve if the ADRP were not surface active (as demonstrated by the surface-tension measurements).

### 3.5. Free-Standing Monolayer Formation

The device used to study phospholipid monolayer was a simplified version of the previously described one and consisted essentially of a cylindrical hole in a PDMS (Sylgard 184) matrix that was bonded to a glass substrate; cf. Figure 1D. Details of the fabrication and chip geometry, can be found in ref. [27].

To produce the triolein–lipid mixture, a specific lipid composition (2% of phospholipids in total weight ratio) was dissolved in chloroform. This solution was then dried under vacuum. Triolein was added to the dried components and mixed in an ultrasonic bath. The resulting phospholipid mixture also contained 2% (in molar ratio) of DOPE-Cy5 as a fluorescent probe. To form a monolayer, 6 μL of the as-prepared triolein–lipid mixture was first placed in the cylindrical opening of the PDMS microchip, and then 20 μL of 150 M KCl buffer was added on top. This buffer also contained 1 μM of fluorescent ADRP. Within seconds, a phospholipid monolayer formed at the triolein–buffer interface, with a composition similar to the lipid composition of the originally prepared triolein solution. All experiments were performed at a room temperature of 23 °C.

### 3.6. Confocal Imaging and FRAP Experiments

Confocal images were acquired with an inverted microscope (Nikon Ti-Eclipse) with an Intensilight Epi-fluorescence light source and a laser unit (LU-NV, Nikon). The confocal microscope was equipped with a Yokogawa spinning disk head (CSU-W1; Andor Technology) and a module for fluorescence recovery after photo-bleaching (FRAPPA; Andor Technology). Confocal imaging was conducted using excitation wavelengths of 481 nm (for Alexa488) and 647 nm (for DOPE-Cy5). The used emission filters had the wavelengths/bandwidths of 525/30 nm and 685/40 nm, respectively. For the FRAP experiments, a pinhole size of 50 μM was used in combination with a 40× oil objective having a working distance of 220 μm. Prior to bleaching, a circular stimulation area with diameter ranging between 5 and 50 μm was selected inside the bilayer (to increase measurement quality). At the beginning of a FRAP measurement, fluorescence imaging was performed for 20 s for each individual experiment. Then, bleaching was performed by increasing the laser power in the stimulation area (defined by the pinhole size) to the maximum laser power for 20 s (70 mW for 481 and 561 nm, 125 mW for 647 nm). During the following fluorescence recovery, image acquisition was continued for at least 5 min to be able to observe the full recovery. The thus obtained fluorescence recovery (FRAP) data were directly fitted with the Soumpasis equation using Python [26,27,34]. All experiments were performed at a room temperature of 23 °C.

### 3.7. Surface Tension Measurement

Surface tensions were determined by the pending-drop method using a contact angle measurement device, an OCA 20 (DataPhysics, Filderstadt, Germany). For this method, a droplet with a defined volume of the denser fluid, here the buffer solution ($\rho_{buffer}$ = 0.998 g/cm${}^{3}$), is created at the tip of a needle, which is immersed in a transparent cuvette filled with the fluid of lesser density—here, the squalene–phospholipid mixture ($\rho_{oil}$ = 0.858 g/cm${}^{3}$). While gravity drags the droplet down, buoyancy and surface forces keep the droplet in place. The surface tension can then be determined based on a Young–Laplace fit to an shadow image of the droplet, which is automatically accomplished by the software SCA 20. For best quality of the fitted data, the droplet volume should be chosen to be as large as possible, yet on the other hand, the detachment droplet volume at a fully lipid-decorated oil–water interface defines an upper limit of the droplet’s size. As an optimum for the considered system, we set the droplet volume to 2.0 ± 0.5 μL.

From the surface tension values obtained from those pendant drop measurements (listed in Table 1), and the bilayer contact angle 2θ obtained from optical micrographs (listed in Table 1), cf. Figure 2C, the bilayer tension can be calculated using Young’s equation:

(3) Γ=2γcos(θ).

All those measured surface tension and contact angle values and those calculated from them are listed in Table 1.

## 4. Conclusions

In this manuscript, we have extended previous works by successfully reconstituting a full-length LD protein (ADRP) in a bilayer-embedded LD. We focused our interests on the influence of ADRPs on the phospholipid diffusion and the 3D shape of bilayer-embedded LDs. We observed that the surface-active ADRP molecules strongly affect the shape of the bilayer-embedded LDs. The traditional expected lens shape is believed to have vanished in favor of a pancake shape as a result of the non-homogeneous protein decoration on the LD’s surface, i.e., a relative enrichment of ADRP molecules close to the three-phase contact line and a depletion at the center of the droplet. This spontaneous asymmetry may facilitate spontaneous droplet bulging and have consequences for LD biogenesis [39]. This specific pancake-like droplet shape was maintained (or even enhanced) for varying surface tension tuned by increasing cholesterol concentration. However, despite the unexpected pancake shape, the contact angles measured locally on the three-phase contact line are in agreement with expectations from wetting theory, assuming contact angles averaged for several droplets and surface tensions obtained from bulk measurements. We think that the unexpected shape of these LDs may affect how they work, how they grow, or how they bulge.

Additionally, we also studied the diffusion properties of phospholipids and ADRP within our system. In particular, we measured that the ADRP molecules can diffuse freely throughout the LD’s surface. Consistently, the phospholipid diffusion properties on a LD surface were very similar to those of a bilayer and were barely affected by the presence of ADRP. This point seems to be in agreement with the literature. However, ADRPs facilitate the transport of phospholipids between a bilayer and a bilayer-embedded LD, presumably by destroying the hypothesized PE-ring around an LD forming the structural origin of the diffusion barrier [26]. These results show that all molecules may diffuse freely, proving that the pancake shape is not the result of a diffusion-related artifact, but rather the consequence of the accumulation of ADRP molecules close to the three-phase contact line.

## Figures and Tables

**Table 1:** Physical characterization of a symmetric bilayer and LDs inserted in it. The phospholipids diffused between the monolayer covering the LD and the bilayer, so the LD monolayer’s phospholipidic composition was the same as that of the bilayer after a long time. LD surface tension $\gamma_{LD}$ was obtained from pendant drop measurements, bilayer tension Γ was obtained from optically determined contact angles θ and $\gamma_{LD}$ using Equation (2), and LD contact angles were obtained from 3D confocal micrographs. Each value was obtained by averaging 30 different measurements.

Protein	Chol	Bilayer	LB Contact	LD Tension	LD Contact	LD Contact
(μM)	(%)	Tension Γ	Angle θ	$\gamma_{\mathrm{LD}}$	Angle $\alpha_{U}$	Angle $\alpha_{L}$
1	0%	2 mN/m	(64 ± 3)${}^{{}^{\circ}}$	1.06 mN/m	(42 ± 3)${}^{{}^{\circ}}$	(35 ± 3)${}^{{}^{\circ}}$
1	20%	2.4 mN/m	(67 ± 3)${}^{{}^{\circ}}$	2.01 mN/m	(65 ± 3)${}^{{}^{\circ}}$	(55 ± 3)${}^{{}^{\circ}}$
1	40%	3.1 mN/m	(69 ± 2)${}^{{}^{\circ}}$	3.9 mN/m	(75 ± 3)${}^{{}^{\circ}}$	(65 ± 3)${}^{{}^{\circ}}$
0	0%	2 mN/m	(63 ± 2)${}^{{}^{\circ}}$	1.6 mN/m	(49 ± 3)${}^{{}^{\circ}}$	(51 ± 3)${}^{{}^{\circ}}$

## Data Availability

The datasets generated during and/or analyzed during the current study are available from the corresponding author on reasonable request.

## Supplementary Materials

The following supporting information can be downloaded at: https://www.mdpi.com/article/10.3390/ijms24032072/s1.
